# Hospitalization for pulmonary embolism associated with antecedent testosterone or estrogen therapy in patients found to have familial and acquired thrombophilia

**DOI:** 10.1186/s12878-016-0045-9

**Published:** 2016-03-08

**Authors:** Marloe Prince, Charles J. Glueck, Parth Shah, Ashwin Kumar, Michael Goldenberg, Matan Rothschild, Nasim Motayar, Vybhav Jetty, Kevin Lee, Ping Wang

**Affiliations:** From the Internal Medicine Residency Program, Cholesterol, Metabolism, and Thrombosis Center of the Jewish Hospital of Cincinnati, 2135 Dana Avenue, Suite 430, Cincinnati, OH 45207 USA

**Keywords:** Thrombophilia, Testosterone, Estrogen, Pulmonary embolus

## Abstract

**Background:**

In patients hospitalized over a 4 year period for pulmonary embolism (PE), we assessed relationships of testosterone (TT) and estrogen therapy (ET) anteceding PE in patients found to have familial-acquired thrombophilia.

**Methods:**

From 2011 through 2014, 347 patients were hospitalized in Cincinnati Mercy Hospitals with PE. Retrospective chart review was used to identify patients receiving TT or ET before PE; coagulation studies were done prospectively if necessary.

**Results:**

Preceding hospitalization for PE, 8 of 154 men (5 %) used TT, and 24 of 193 women (12 %) used ET. The median number of months from the initiation of TT or ET to development of PE was 7 months in men and 18 months in women. Of the 6 men having coagulation measures, all had ≥ 1 thrombophilia, and of the 18 women having measures of coagulation, 16 had ≥ 1 thrombophilia. The sensitivity of a previous history of thrombosis to predict PE was low, 25 % (2/8 men), 4 % (1/24 women).

**Conclusions:**

Of 154 men hospitalized for PE, 8 (5 %) used TT, and of 193 women, 24 (12 %) used ET. Our data suggests that PE is an important complication of TT in men and ET in women, in part reflecting an interaction between familial and acquired thrombophilia and exogenous hormone use.

## Background

When testosterone therapy (TT) is given to men or women with underlying familial or acquired thrombophilia, venous thromboembolism (VTE), deep venous thrombosis (DVT), pulmonary emboli (PE), ocular thrombosis, and osteonecrosis may occur [1–8]. In 67 recently reported cases of VTE, Glueck et al. [8] compared thrombophilia in 67 cases (59 men and 8 women) with thrombotic events after starting testosterone therapy versus 111 patient controls having unprovoked venous thrombotic events without TT. In the 67 patients, thrombosis (47 deep venous thrombosis-pulmonary embolism, 16 osteonecrosis, and 4 ocular thrombosis) occurred 6 months (median) after starting TT. Cases differed from controls for factor V Leiden heterozygosity (16 of the 67 [24 %] vs 13 [12 %] of the 111, *P* = .038) and for lupus anticoagulant (9 [14 %] of the 64 vs 4 [4 %] of the 106, *P* = .019). After a first thrombotic event and continuing TT, 11 cases had a second thrombotic event, despite adequate anticoagulation, 6 of whom, still anticoagulated, had a third thrombosis. Screening for thrombophilia before starting TT should identify men and women at high risk for thrombotic events with an adverse risk-benefit ratio for TT. Glueck et al. [8] concluded that when TT is given to patients with familial and acquired thrombophilia, thrombosis may occur and recur in thrombophilic men despite anticoagulation.

About 10 % of patients with symptomatic DVTs develop severe post thrombotic syndrome within 5 years [9]. Despite adequate treatment, up to 25 % of patients with symptomatic DVT-PE have recurrent VTE within 5 years [10]. Moreover, 25 % of patients with VTE do not survive the first year after diagnosis [11].

In 596 men hospitalized for DVT-PE, we previously reported that 7 (1.2 %) had taken TT before and at time of their admission [3]. Of these 7 men, all 5 who had evaluation of thrombophilia-hypofibrinolysis were found to have previously undiagnosed procoagulants. Separately, we studied 147 men hospitalized for DVT-PE, finding 2 (1.4 %) with antecedent TT use [5]. Both men had previously undiagnosed thrombophilia [5]. Parallel to the TT-VTE relationship in men, estrogen-progestin birth control pills (BCP) and hormone replacement therapy (HRT) in women are associated with VTE [12–14], and the issue of screening for thrombophilia before prescription of BCP or HRT is contentious because of cost-effectiveness issues [15].

In 347 patients (154 men, 193 women) hospitalized over a 4-year period for pulmonary embolism, we investigated testosterone and estrogen therapy (ET) use anteceding PE, and studies of procoagulants in TT and ET users.

## Methods

### Patients

#### Ethics, consent

The study was carried out following a protocol approved by the Jewish Hospital Institutional Review Board, with signed informed consent. No identifiable clinical data is presented.

From 2011 through 2014, using review of electronic medical records, 347 patients were hospitalized in Cincinnati Mercy Hospitals with PE. Only patients hospitalized for PE were studied. Retrospective chart review was used to document TT or ET use anteceding PE. We prospectively performed coagulation studies in those patients who used TT or ET, not having previous evaluation of thrombophilia-hypofibrinolysis.

Retrospectively, we reviewed coagulation data obtained at hospitalization for PE from electronic medical records in two other groups of patients not exposed to TT or ET, 78 with cancer associated with PE (17 had coagulation data), and 237 free of cancer (116 had coagulation data).

### Studies of thrombophilia and hypofibrinolysis

PCR measures of the Factor V Leiden, prothrombin, MTHFR mutations and serologic measures of activated protein C resistance [16, 17], antigenic proteins antithrombin III, C, total S, free S, homocysteine, factors VIII and XI [18, 19], the lupus anticoagulant, and anticardiolipin antibodies were carried out using established methods [20–22].

PCR measures of the 4G/5G mutation in the plasminogen activator inhibitor (Serpine1) gene [8, 23–25] were carried out using established methods [20–22]. Serologic studies of plasminogen activator inhibitor activity were not done.

### Statistical methods

Within gender, users and non-users of ET/TT were compared by Fisher’s exact test and by Wilcoxon tests. Thrombophilia in the 24 hormone users (6 men on TT, 18 women on ET) was compared to 116 cases (62 men, 54 women) with PE, not receiving TT or ET and free of cancer, and to 17 cases (9 men, 8 women) with cancer, not receiving TT or ET by Fisher’s exact test.

## Results

**Fig. 1:**
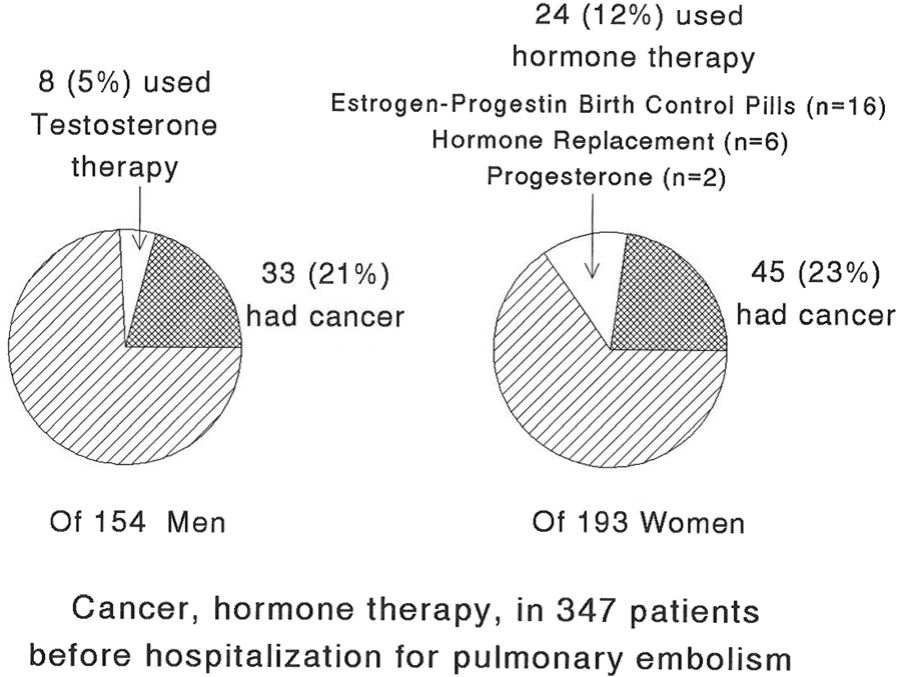
Percent of 154 men admitted with pulmonary embolism (PE) who used testosterone therapy before PE, and the percent who had cancer associated with PE. Percent of 193 women admitted with pulmonary embolism (PE) who used estrogen-progestin birth control pills (*n* = 16), estrogen-progestin hormone replacement therapy (*n* = 6) or progesterone (*n* = 2) before PE, and percent who had cancer associated with PE

Preceding hospitalization for PE, of the 154 men and 193 women, 8 men (5 % of men) used TT, 24 women (12 % of women) used ET (16 estrogen-progestin birth control pills [BCP], 6 hormone replacement therapy [HRT], 2 progesterone), Fig. 1, Table 1. From the initiation of TT or ET to development of PE, the median time was 7 months in men and 18 months in women.

**Table 1:** Demographics in 347 pulmonary embolism patients (193 women, 154 men), by gender and by hormone use

Women			
	Hormone user, *n* = 24 (12 %)	Non user, *n* = 169 (88 %)	all women *n* = 193 (100 %)
Age (year)	42 ± 13, median 38****	66 ± 17, median 69	63 ± 18, median 65
Smoking	7/24 (29 %)*	96/169 (57 %)	103/193 (53 %)
Hormone	BC pill 16 (67 %) Estrogen 6 (25 %) Progesterone 2 (8 %)		
Cancer	0/24 (0 %)**	45/169 (27 %)	45/193 (23 %)
DM	2/24 (8 %)	42/169 (25 %)	44/193 (23 %)
Death	0/24 (0 %)	3/169 (2 %)	3/193 (2 %)
Thrombosis history	1/24 (4 %)*	40/168 (24 %), 1 missing	41/193 (21 %)
Men			
	Hormone user, *n* = 8 (5 %)	Non user *n* = 146 (95 %)	All men *n* = 154 (100 %)
Age (year)	48 ± 23, median 56	63 ± 17, median 65	62 ± 17, median 64
Smoking	5/8 (63 %)	103/146 (71 %)	108/154 (70 %)
Hormone	Testosterone		
Cancer	0/8 (0 %)	33/146 (23 %)	33/154 (21 %)
DM	2/8 (25 %)	31/146 (21 %)	33/154 (21 %)
Death	0/8 (0 %)	2/146 (1 %)	2/154 (1 %)
Thrombosis history	2/8 (25 %)	31/146 (21 %)	33/154 (21 %)

* *p* < .05, ** *p* < .01, comparing hormone user vs non user in each gender by Fisher’s exact test

**** *p* < .0001, comparing hormone user vs non user in each gender by Wilcoxon test

Median age in the 8 men was 56, marginally (*p* = .068) younger than in the 146 men not on TT (65). Median age in the 24 women with PE on ET was 38, younger than 169 women not taking ET (69, *p* < .0001), Table 1. Women taking ET with subsequent PE were less likely than women not taking ET to have cancer, and were less likely to have a previous thrombosis history (4 % vs 24 %, *p* < .05), Table 1.

Of the 8 men using TT before their PE, 6 used TT gels, 50 mg/day, and 2 had intra muscular TT 50 mg/week. Of these 8 men, 5 (63 %) smoked, 2 had a previous history of thrombotic events, and 2 had type 2 diabetes, Table 1.

Coagulation evaluations were done in 6 of the 8 men with TT anteceding PE, Table 2. All 6 had ≥ 1 thrombophilia or hypofibrinolysis: 1 heterozygous for the G20210A prothrombin gene mutation, 1 homozygous for the 4G4G PAI-1 gene mutation, 1 with high factor VIII, 1 with high ACLA IgG, 3 with high homocysteine (1 of whom had MTHFR C677T homozygosity), 2 with low protein C, 2 with low protein S, and 2 with low free protein S, Table 2. Two of 8 men had Klinefelters syndrome.

**Table 2:** Abnormal measures of thrombophilia and hypofibrinolysis in 8 men who using testosterone therapy before pulmonary embolism (6 men had coagulation measures)

	PTG	MTHFR	PAIG	Homocysteine	Factor VIII	ACLA IgG	Pro C	PRO S	Free S
Abnormal range	TC/TT	TT	4G4G	Dated cut point^a^	> 150 %	Dated cut point^b^	< 73 %	< 63 %	< 66 %
ID# 1				12.0			61		
2	TC					18.0			57
4								64	
5			4G4G		223		47	49	43
6		TT		17.3					
7				11.0					

^a^dated cut point for Homocysteine high: ≥ 15 (11/15/08-12/2/14); ≥ 10.4 (after 12/3/14)

^b^dated cut point for IgG high: ≥ 23 GPL (before 10/31/12); ≥ 15 (after 11/1/12)

Of the 24 women taking ET before PE, 2 were diabetic, 1 had a previous history of thrombosis and 7 (29 %) smoked, Table 1.

Of the 24 women, 18 had measures of coagulation, and 16 (89 %) had ≥ 1 thrombophilia (Table 3). Four women were V Leiden heterozygotes, 1 prothrombin gene heterozygote, 2 had high Factor VIII, 1 had high Factor XI, 2 were positive for the lupus anticoagulant, 3 had low protein S, 2 had low Free S, 3 had low antithrombin III, and 3 had high ACLA, Table 3.

**Table 3:** Abnormal measures of thrombophilia and hypofibrinolysis in 24 women who used hormone replacement therapy on estrogen-progestin oral contraceptives before pulmonary embolism (18 women had coagulation measures)

	Factor V	PTG	Factor VIII	Factor XI	ACLA IgG	ACLA IgM	Lupus anticoagulant	Pro S	Free S	Anti III
Abnormal range	TC/TT	TC/TT	> 150 %	> 150 %	Dated cut point^a^		Y	< 63 %	< 66 %	< 80 %
ID# 1										78
3						20.0				
4										
5	TC							47		
6										70
8	TC									
9							Y			71
10			212							
11							Y			
12									61	
13					16.0			59		
14										
15			301	151		17.0			39	
17		TC								
18								48		
22	TC									
24	TC									

^a^dated cut point for IgG high: ≥ 23 GPL (before 10/31/12); ≥ 15 (after 11/1/12)

dated cut point for IgM high: ≥ 10 MPL (before 4/30/12); ≥ 13 (after 5/1/12)

Cancer was associated with PE in 78 patients, 22 % of the cohort (45 women [23 % of women], 33 men [21 % of men], Table 1, Fig. 1). None of the cancer patients took either TT or ET, Table 1. Of the 33 men and 45 women with PE and concurrent cancer (Fig. 1), hospital based coagulation measures (Factor V Leiden, homocysteine, lupus anticoagulant, anticardiolipin antibody IgG and IgM, proteins C, S, and antithrombin III) were obtained in 17 (9 men, 8 women, Tables 4 and 5). Thrombophilia was rare in the 17 cancer patients with PE, with exception of homocysteine, which was high in 40 % of cancer patients, marginally more common than in the 24 hormone users (13 %, *p* = .063), Tables 4 and 5.

**Table 4:** Coagulation disorders in 24 cases (6 men [on testosterone] and 18 women [on estrogen]) who had testosterone/hormone therapy before PE, compared to 116 cases with PE but no hormone, no cancer (62 men, 54 women), and compared to 17 cancer cases (9 men, 8 women)

	Factor V	PTG	MTHFR	PAIG	Homocys-teine^a^	Lupus anticoagulant	ACLA IgG	ACLA IgM
Abnormal range	TC,TT	TC,TT	TT	4G4G	umol/l	Positive	Dated^b^	Dated^c^
Hormone Cases (*n* = 24, 6 men on TT, 18 women on ET)	4/24 (17 %)	2/24 (8 %)	1/24 (4 %)	1/24 (4 %)	3/24 (13 %)	2/24 (8 %)	2/24 (8 %)	2/24 (8 %)
PE_no hormone, no Cancer (*n* = 116, 62 men, 54 women)	15/105 (14 %)	7/71 (10 %)			18/43 (42 %) **	17/68 (25 %)	1/61 (2 %)	5/58 (9 %)
Cancer (*n* = 17, 9 men, 8 women)	0/15 (0 %)				6/15 (40 %)	1/17 (6 %)	0/15 (0 %)	1/17 (6 %)

**p < .025, comparing with Hormone cases by Fisher’s test

^a^dated cut point for Homocysteine high: ≥ 15 (11/15/08-12/2/14); ≥10.4 (after 12/3/14)

^b^dated cut point for IgG high: ≥ 23 GPL (before 10/31/12); ≥ 15 (after 11/1/12)

^c^dated cut point for IgM high: ≥ 10 MPL (before 4/30/12); ≥ 13 (after 5/1/12)

**Table 5:** Coagulation disorders in 24 cases (6 men [on testosterone] and 18 women [on estrogen]) who had testosterone/hormone therapy before PE, compared to 116 cases with PE but no hormone, no cancer (62 men, 54 women), and compared to 17 cancer cases (9 men, 8 women)

	Factor VIII	Factor XI	Protein C	Protein S	Free S	Antithrombin III
Abnormal range	> 150 %	> 150 %	< 73 %	< 63 %	< 66 %	< 80
Hormone Cases (*n* = 24, 6 men on TT, 18 women on ET)	3/24 (13 %)	1/23 (4 %)	2/24 (8 %)	5/24 (21 %)	4/24 (17 %)	3/24 (13 %)
PE_no hormone, no Cancer (*n* = 116, 62 men, 54 women)			27/90 (30 %)*	8/77 (10 %)	11/74 (15 %)	8/80 (10 %)
Cancer (*n* = 17, 9 men, 8 women)			1/16 (6 %)	1/16 (6 %)		1/14 (7 %)

* *p* < .05, comparing with Hormone cases by Fisher’s test

Of the 237 patients hospitalized with PE, free of cancer and free of TT or ET supplementation, 116 had thrombophilia measures, Tables 4 and 5. These 116 cases did not differ (p > 0.4) from the 24 hormone using cases, except for high homocysteine (42 % vs 13 %, *p* < .025) and low protein C (30 % vs 8 %, *p* < .05), Tables 4 and 5.

## Discussion

Increased risk of VTE in women using combined oral contraceptives has been known for at least 52 years [26], and is well recognized for hormone replacement therapy [27]. By comparison, the association of VTE in men [1] and women [4, 6] using TT has only recently been described, as of 2011 and 2013–2015 respectively [1–6, 8]. Previously, in 596 men hospitalized for DVT-PE, we reported that 7 (1.2 %) had taken TT before and at time of their admission [3], and all 5 men who had evaluation of thrombophilia-hypofibrinolysis were found to have previously undiagnosed procoagulants. Separately, we studied 147 men hospitalized for DVT-PE, finding 2 (1.4 %) with antecedent TT use, both of whom had previously undiagnosed thrombophilia [5].

In the current study, of 154 men hospitalized for PE, 8 (5 %) used TT, and of 193 women, 24 (12 %) used ET. congruent with previous studies [26, 27]. Congruent with our previous reports [3, 5], all of the men with PE after TT in the current study were found to have familial-acquired thrombophilia, as were 16/18 women (89 %) with PE after ET. Thrombophilia in the 24 cases using TT and ET was comparable to that in 116 cases not using TT or ET. As in the current study, when TT or ET are given to patients with previously undiagnosed thrombophilia, thrombosis commonly occurs [8].

In the current study, thrombophilia was rare in patients whose PE was associated with cancer, in agreement with the report by Fiaz et al. [28] who reported that thrombophilia was more common among VTE patients without cancer than in those with cancer.

Health [9, 10] and cost [29–31] ramifications of a PE, either unprovoked or after TT or ET are significant. After PE, there is a high cost of hospitalization, re-hospitalization, and post-PE care [29]. As summarized by Reitsma [32], “…the one-year mortality is 20 % after a first VTE. Of the surviving patients, 15–25 % will experience a recurrent episode of VTE in the three years after the first event. Primary and secondary prevention is key to reducing death and disability from VTE.”

Selective coagulation screening based on prior VTE history, if applied to our 347 patients hospitalized for PE, had low sensitivity, and would have identified only 25 % of men with PE on TT, and only 4 % of women with PE on estrogen-progestin oral contraceptives-HRT. However, cost-effectiveness studies [33, 34] suggest that selective coagulation screening based on prior VTE history is more cost-effective than universal screening.

Limitations of our study include its retrospective observational nature, and a limited sample size of patients who took TT or ET and had coagulation screening. Our study is further limited by not having coagulation data on patients admitted with PE who died before coagulation measures were obtained.

## Conclusions

Of 154 men hospitalized for PE, 8 (5 %) used TT, and of 193 women, 24 (12 %) used ET. Our data suggests that PE is an important complication of TT in men and ET in women, in part reflecting an interaction between familial and acquired thrombophilia and exogenous hormone use.

Our findings may have important clinical ramifications, because VTE risk is an important determinant of the benefit risk ratio of both TT and ET. In women, PE accounts for about one third of the incidence of potentially fatal VTE events associated with HRT [35], and HRT increases the risk of VTE by 2- to 3-fold [36].

Reitsma [32] has concluded that “…for primary prevention of VTE, genetic testing is not likely to play a role in the future.” However, as the cost of screening for familial and acquired thrombophilias falls over time, with the development of multilocus genetic risk scores to improve classification, we believe that coagulation studies before starting TT, ET, and oral contraceptive therapy should be done as an approach to primary prevention of VTE, including at least PCR studies of the Factor V Leiden and Prothrombin gene mutations, Factors VIII and XI, homocysteine, and the lupus anticoagulant.

## Availability of supporting data

All supporting data is available in SAS and Excel files from Ping Wang PhD (pxwang@mercy.com).

## Abbreviations

PE: Pulmonary embolism
TT: Testosterone therapy
ET: Estrogen therapy
VTE: Venous thromboembolism
DVT: Deep venous thrombosis
BCP: Birth control pills
HRT: Hormone replacement therapy
ACLA: Anticardiolipin antibodies
